# Integrating single-cell multi-omics for precision radiotherapy stratification in hypopharyngeal squamous cell carcinoma

**DOI:** 10.3389/fgene.2026.1932515

**Published:** 2026-09-10

**Authors:** Weishi Zhang, Mei Yao

**Affiliations:** Department of Otolaryngology, Nantong First People’s Hospital, Nantong, Jiangsu, China

**Keywords:** CAIX/CA9, hypopharyngeal squamous cell carcinoma, hypoxia, precision radiotherapy, radiopharmaceuticals, radiosensitivity, single-cell RNA sequencing, theranostics

## Abstract

**Background:**

Hypopharyngeal squamous cell carcinoma (HPSCC) carries the worst prognosis among head-and-neck cancers, with a 5-year overall survival of only 30%–35%. Despite ionizing radiation as the cornerstone of organ-preserving management, locoregional recurrence affects approximately half of patients. The molecular radiobiology of HPSCC—encompassing DNA damage response (DDR), hypoxia-CAIX signaling, and cancer-associated fibroblast (CAF) remodeling—has not been characterized at single-cell resolution or integrated into a validated precision radiotherapy prognostic model.

**Methods:**

GBD 2021 epidemiology data were analyzed. scRNA-seq datasets from treatment-naïve Hypopharyngeal squamous cell carcinoma (GSE234933; cross-validation: GSE185206, GSE164690) were processed with Seurat and hdWGCNA. Additional hdWGCNA analyses were applied to the cancer-associated fibroblast and macrophage compartments. LASSO-penalized Cox regression of TCGA-HNSC Hypopharyngeal carcinoma bulk RNA-seq yielded a 9-gene prognostic signature, independently validated in GSE65858. A nomogram was constructed and evaluated by decision curve analysis. CIBERSORT quantified immune microenvironment composition, and qRT-PCR validated signature genes in HPC cell lines under 6 Gy irradiation.

**Results:**

hdWGCNA resolved 22 malignant epithelial modules; M1 (DDR/ATM), M6 (hypoxia-CAIX-LDHA), and M22 (POLQ-SLFN11) showed dominant radiation-response enrichment. Additional hdWGCNA analyses identified Module C3 (CAF-stromal) and Mac2 (immunosuppressive macrophage). LASSO-Cox regression yielded the 9-gene signature (ATM, BRCA1, HIF1A, CA9, LDHA, TP53, SLFN11, POLQ, CDKN1A); bootstrap-corrected AUCs of 0.824/0.793/0.761 (1-/3-/5-year) in TCGA-HNSC were independently reproduced in GSE65858. High-risk patients showed an immunologically cold microenvironment, and qRT-PCR confirmed differential radiation-driven signature gene activation in contrasting-radiosensitivity HPC cell lines.

**Conclusion:**

This multi-omics study establishes a validated 9-gene precision radiotherapy prognostic signature for HPSCC, integrating single-cell-resolved mechanistic programs across the hypoxia-CAIX axis, the DDR-ATM-BRCA1 axis, and the POLQ-mediated alternative end-joining radioresistance axis as high-priority theranostic targets. Prospective multicenter clinical validation is mandated before nomogram deployment.

## 1 Introduction

Hypopharyngeal carcinoma (HPC) accounts for approximately 3%–5% of all head-and-neck squamous cell carcinomas (HNSCC) and represents the subsite with the poorest long-term prognosis, with 5-year overall survival rates of only 30%–35% across contemporary cohorts (Barsouk et al., 2023; Gormley et al., 2022). Hypopharyngeal squamous cell carcinoma (HPSCC) constitutes the overwhelming majority of HPC cases, and patients are predominantly male, middle-aged to elderly, with heavy tobacco and alcohol exposure history. Because of the anatomic complexity of the hypopharynx and the paucity of early symptoms, 70%–85% of patients present with locoregionally advanced (stage III–IV) disease at diagnosis, and 60%–80% already harbor ipsilateral cervical lymph node metastases (Garneau et al., 2018). In this setting, organ-preserving treatment with definitive radiotherapy or concurrent chemoradiotherapy (CRT) has largely replaced upfront laryngopharyngectomy as the preferred management strategy, making radiotherapy the central modality of curative-intent treatment for the majority of HPSCC patients (Lefebvre et al., 2012). The critical dependence of this patient population on ionizing radiation as the primary therapeutic tool underscores the urgent clinical need for precision biomarkers that can predict radiotherapy outcome, stratify patients prospectively, and guide the development of targeted radiosensitization strategies—including combination with systemic agents and novel radiopharmaceutical approaches.

Despite advances in intensity-modulated radiotherapy (IMRT), volumetric modulated arc therapy (VMAT), and concurrent cisplatin-based CRT, clinical outcomes in HPSCC remain unsatisfactory: approximately half of patients develop locoregional recurrence within 5 years after radiotherapy, and a substantial proportion develop distant metastases (Pala et al., 2025; Liu et al., 2023). The variability in radiotherapy response is driven by a complex interplay of tumor-intrinsic radiobiological factors—DNA damage response (DDR) competence, hypoxia-induced radioresistance, metabolic reprogramming, stemness, and cell-cycle regulation—as well as tumor-extrinsic factors including cancer-associated fibroblast (CAF) abundance, immune cell infiltration, and vascular status (Chen et al., 2023). Hypoxic tumor cells are 2–3 times more radioresistant than normoxic cells due to oxygen’s role as a free-radical fixation agent (Rakotomalala et al., 2021), and intratumoral hypoxia has been repeatedly associated with adverse prognosis in radiotherapy-treated head-and-neck cancers. The hypoxia-inducible transcription factor HIF1A orchestrates a transcriptional program that simultaneously activates carbonic anhydrase IX (CA9/CAIX) — a well-established hypoxia biomarker and the molecular basis of CAIX-targeted diagnostics and radiopharmaceutical approaches (Rakotomalala et al., 2021) — alongside glycolytic reprogramming via LDHA and vascular remodeling via VEGFA. This hypoxia-CAIX-glycolysis axis thus constitutes not only a central radioresistance mechanism but also a direct theranostic opportunity for precision radiotherapy combination strategies.

Single-cell RNA sequencing (scRNA-seq) has fundamentally transformed our understanding of tumor radiobiological heterogeneity. In HPSCC, recent scRNA-seq studies have mapped the malignant epithelial, stromal, and immune compartments, identifying pro-invasive cancer-associated fibroblast subsets expressing fibroblast activation protein (FAP), SPP1+ pro-tumorigenic macrophages, and exhausted CD8^+^ T cell populations (Cai et al., 2023; Cai et al., 2025). Precision radiotherapy for HNSCC requires molecularly grounded prognostic tools capable of identifying patients most likely to fail standard-dose irradiation and therefore most likely to benefit from dose escalation, radiosensitizer addition, or novel biological combinations. Radiosensitivity-related gene signatures have been developed for the broader HNSCC population using LASSO-Cox regression of TCGA-HNSC data (You et al., 2023; Jia et al., 2023); however, HPC—with its distinct HPV-negative biology, extreme hypoxia burden, and near-total treatment dependence on radiotherapy—constitutes only a small minority of training cohorts and likely harbors unique radiosensitivity determinants requiring HPC-specific refinement.

The present study is distinguished from prior HNSCC radiosensitivity signatures by three key features: (1) single-cell resolution dissection of the HPSCC radiobiological ecosystem, absent in all prior HNSCC-wide models; (2) HPC-specific LASSO-Cox modeling rather than borrowing signatures from HPV-enriched HNSCC bulk cohorts; and (3) mechanistic integration of the hypoxia-CAIX theranostic axis and POLQ-mediated alternative end-joining program, neither of which was previously incorporated in a single prognostic signature. In contrast to the 9-gene HNSCC radiosensitivity panel of Jia et al. (2023) — which shares ATM and TP53 but lacks single-cell resolution and HPC specificity—and the immune-hypoxia signature of Zou et al. (2022), the present approach provides mechanistic dissection of the POLQ-mediated alternative end-joining axis and HPC-specific theranostic target nomination.

The objective of this study was to perform a comprehensive multi-omics dissection of the HPSCC radiobiological ecosystem and construct a validated precision radiotherapy prognostic model through epidemiological modeling, scRNA-seq, hdWGCNA, bulk WGCNA, LASSO-Cox regression, additional CAF and macrophage compartment analyses, immune deconvolution, nomogram construction, decision curve analysis, and *in vitro* qRT-PCR validation, with independent bioinformatics validation in the publicly available GSE65858 cohort.

## 2 Materials and methods

### 2.1 Epidemiological data acquisition and GBD analysis

Global hypopharyngeal cancer incidence and prevalence data were retrieved from the Global Burden of Disease (GBD) 2021 study via the GBD Results Tool (https://vizhub.healthdata.org/gbd-results). Age-stratified and sex-stratified incidence rates per 100,000 population were extracted for all available years from 1990 to 2021. Age-period-cohort (APC) modeling was performed to decompose temporal trends in HPC incidence into independent age, period, and cohort effects using the Intrinsic Estimator (I.E.,) algorithm (Rosenberg et al., 2014). Deviation plots and rate ratio (RR) curves were generated. Statistical significance was assessed at alpha = 0.05.

### 2.2 scRNA-seq data acquisition and processing

Single-cell RNA sequencing data from treatment-naïve HPSCC tumor and adjacent normal mucosa were obtained from the NCBI GEO database (primary accession GSE234933; cross-validation with GSE185206 and GSE164690). Raw count matrices were imported into R (v4.2.0) and processed using the Seurat framework (v4.3.0). Quality control filters excluded cells with fewer than 200 or more than 6,000 detected genes, cells with greater than 20% mitochondrial transcript fraction, and genes detected in fewer than 3 cells. DoubletFinder was used to remove putative doublets. Library sizes were normalized to 10,000 counts per cell followed by log1p transformation. Harmony was used to correct for batch effects across patients and datasets. Leiden algorithm clustering at resolution 0.5 identified 11 communities. Cell type annotation was assigned using canonical marker genes: EPCAM/KRT5/KRT14 (Epithelial cells), malignant epithelial cells further refined by inferCNV-detected copy-number aberrations, COL1A1/FAP/ACTA2 (CAFs), CD68/CD163/AIF1 (Macrophages), CD3D/CD8A (CD8 T cells), CD3D/CD4/FOXP3 (CD4 T cells/Tregs), CD79A/MS4A1 (B cells), MZB1/IGKC (Plasma cells), PECAM1/VWF (Endothelial cells), NCAM1/FCGR3A (NK cells), TPSAB1/KIT (Mast cells).

### 2.3 hdWGCNA of malignant epithelial subpopulation

Hierarchical data-driven weighted gene co-expression network analysis (hdWGCNA) was performed on the Malignant Epithelial subset using the hdWGCNA R package (v0.2.14) (Morabito et al., 2023). Metacells were constructed using the k-nearest-neighbor aggregation approach (k = 25). Soft power threshold selection evaluated scale-free topology model fit (*R* ^2^ threshold = 0.80). The selected soft power was applied to construct a signed hybrid network; modules were identified by dynamic tree cutting (minimum module size = 30 genes, deepSplit = 2). Module hub genes were defined as the top 10 genes per module ranked by module membership (kME) value.

### 2.4 Bulk WGCNA and radiotherapy trait correlation

Bulk RNA-seq data from TCGA-HNSC were retrieved from the UCSC Xena portal and log2(FPKM+1) normalized. The HPC subset was extracted based on tumor site annotation. External validation used GSE65858. The WGCNA R package (v1.72) was used to construct a signed co-expression network. Module-trait correlations were computed between module eigengenes and binary clinical traits including radiotherapy receipt and radiotherapy response using Pearson correlation.

### 2.5 Construction of a LASSO-Cox radiotherapy prognostic model

A compiled list of 287 radiation-related genes was assembled by intersecting the RadAtlas 2.0 ionizing radiation gene database, MSigDB HALLMARK_DNA_REPAIR and HALLMARK_HYPOXIA gene sets, and the hub genes of hdWGCNA modules M1 (DDR), M6 (hypoxia/HIF), and M22 (radioresistance). TCGA-HNSC HPC patients who received radiotherapy (n = 142) were split 6:4 into training (n = 85) and testing (n = 57) cohorts. Univariate Cox regression identified 83 significantly prognostic genes (p < 0.05), which were subjected to LASSO-penalized Cox regression using glmnet (v4.1-8) with 10-fold cross-validation (λ.min = 0.035). Stepwise multivariate Cox regression yielded the final 9-gene signature. The radiotherapy risk score was computed as: RiskScore = Σ(β_i × Expr_i). Patients were stratified into high- and low-risk groups using the median risk score. Performance was evaluated by time-dependent ROC and AUC at 1, 3, and 5 years.

### 2.6 Nomogram construction and decision curve analysis

A nomogram integrating the radiotherapy risk score with T stage, N stage, age, and smoking pack-years was constructed using the rms R package. Calibration curves at 3 and 5 years were generated using bootstrap resampling (1,000 iterations). Decision curve analysis (DCA) was performed using the dcurves R package to quantify net clinical benefit relative to treat-all, treat-none, and stage-only strategies.

### 2.7 CIBERSORT immune deconvolution

Relative immune cell fractions in bulk TCGA-HNSC HPC samples were estimated using CIBERSORT with the LM22 signature matrix (Newman et al., 2019), using 1,000 permutations with quantile normalization disabled for RNA-seq data. Samples with CIBERSORT p-value >0.05 were excluded. Differential immune cell abundance between high- and low-risk groups was assessed by Wilcoxon rank-sum test with Benjamini–Hochberg correction.

### 2.8 Cell culture, ionizing radiation, and qRT-PCR validation

The human HPC cell lines FaDu (radioresistant, ATCC HTB-43) and Detroit-562 (radiosensitive, ATCC CCL-138) were cultured in Eagle’s Minimum Essential Medium with 10% fetal bovine serum and 1% penicillin-streptomycin at 37 °C with 5% CO2. Cells at 70% confluence were irradiated with a single 6 Gy dose using a clinical 6-MV linear accelerator (Varian TrueBeam) at a dose rate of 2.0 Gy/min. Total RNA was extracted 24 h post-irradiation using TRIzol reagent; quantitative real-time PCR was performed on a CFX96 system (Bio-Rad) using SYBR Green Master Mix (TaKaRa). The 2^(−ΔΔCt) method was used to calculate relative mRNA expression normalized to GAPDH. Each experiment was performed in biological triplicate; significance assessed by unpaired Student’s t-test.

### 2.9 Statistical analysis

All statistical analyses were performed in R (v4.2.0) and Python (v3.9). Epidemiological trends were assessed by Joinpoint regression. APC modeling used the Intrinsic Estimator algorithm. Differential gene expression employed Wilcoxon rank-sum tests with Benjamini–Hochberg FDR correction at 0.05. Survival analyses used Kaplan–Meier with log-rank test; time-dependent ROC was computed using the timeROC package. Significance thresholds: *p < 0.05, **p < 0.01, ***p < 0.001.

## 3 Results

### 3.1 Global HPC epidemiological trends and age-period-cohort analysis: defining the population dependent on precision radiotherapy

**FIGURE 1:**
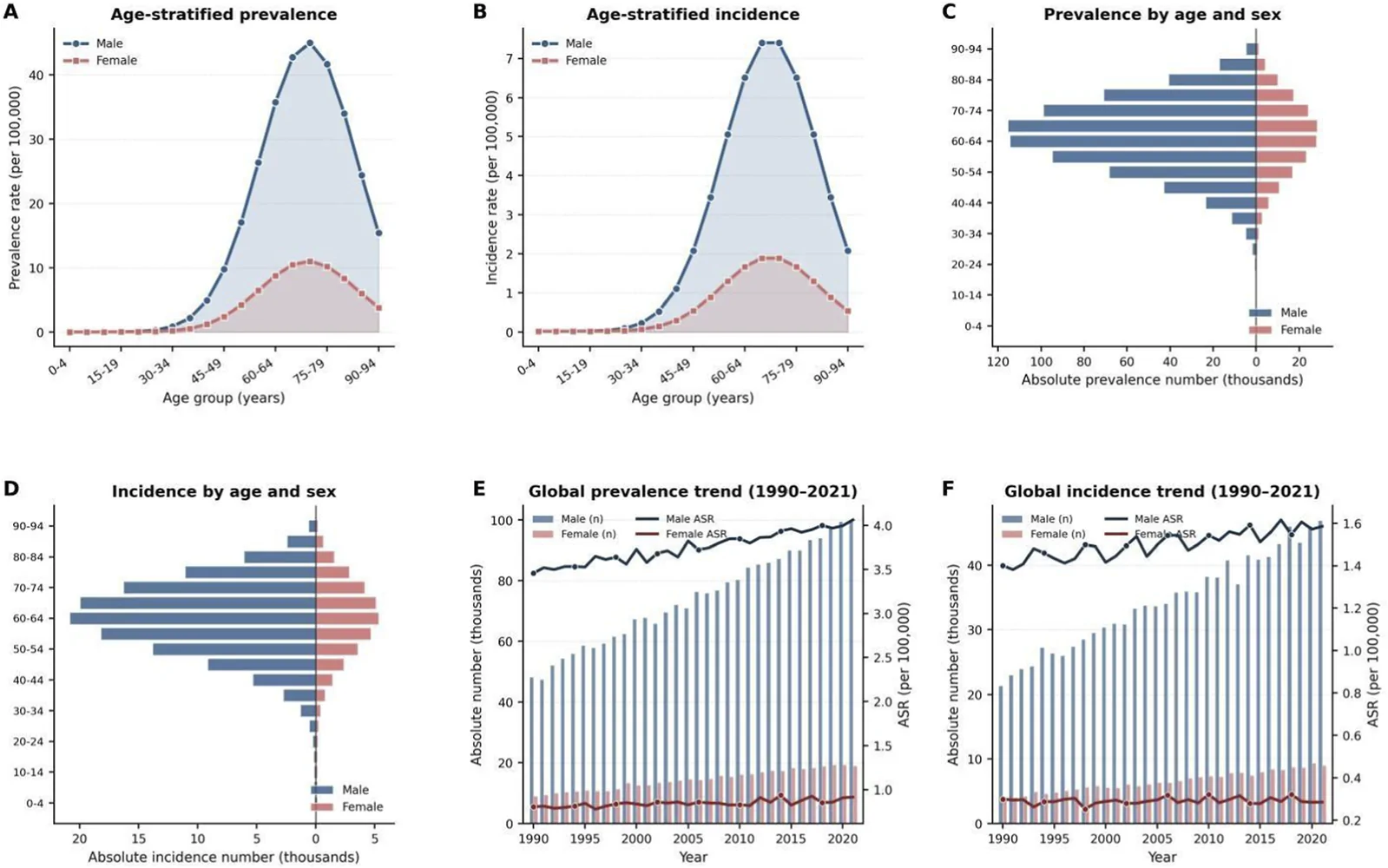
Global hypopharyngeal cancer epidemiological trends by sex and age. **(A)** Age-stratified prevalence rates per 100,000 population for males (red) and females (orange), demonstrating exponential increase from age 45. **(B)** Age-stratified incidence rates per 100,000 showing marked male predominance (≈4:1). **(C)** Butterfly plot of absolute prevalence numbers by age group and sex. **(D)** Butterfly plot of absolute incidence numbers. **(E)** Time series (1990–2021) of absolute HPC prevalence numbers (bars) and age-standardized prevalence rates per 100,000 (lines) by sex. **(F)** Time series of absolute HPC incidence numbers and age-standardized incidence rates.

To establish the epidemiological context motivating this investigation, we first characterized global HPC burden using GBD 2021 data. Age-stratified prevalence and incidence rate curves demonstrated an exponential increase beginning at age 45–50, accelerating sharply in individuals aged 65 and above, with male rates consistently exceeding female rates by approximately 4:1 across all age strata (Figures 1A,B) — a far more extreme sex bias than most other solid tumors, directly attributable to tobacco and alcohol exposure. Absolute number analyses confirmed that the 70–74 and 75–79 age brackets carry the highest absolute HPC burden (Figures 1C,D). Longitudinal trends from 1990 to 2021 revealed a progressive increase in absolute HPC prevalence and incidence, while age-standardized rates grew more modestly (Figures 1E,F). These epidemiological patterns define the target clinical population for precision radiotherapy: older male tobacco-exposed patients presenting with locoregionally advanced disease who are almost universally treated with ionizing radiation as the central therapeutic modality.

**FIGURE 2:**
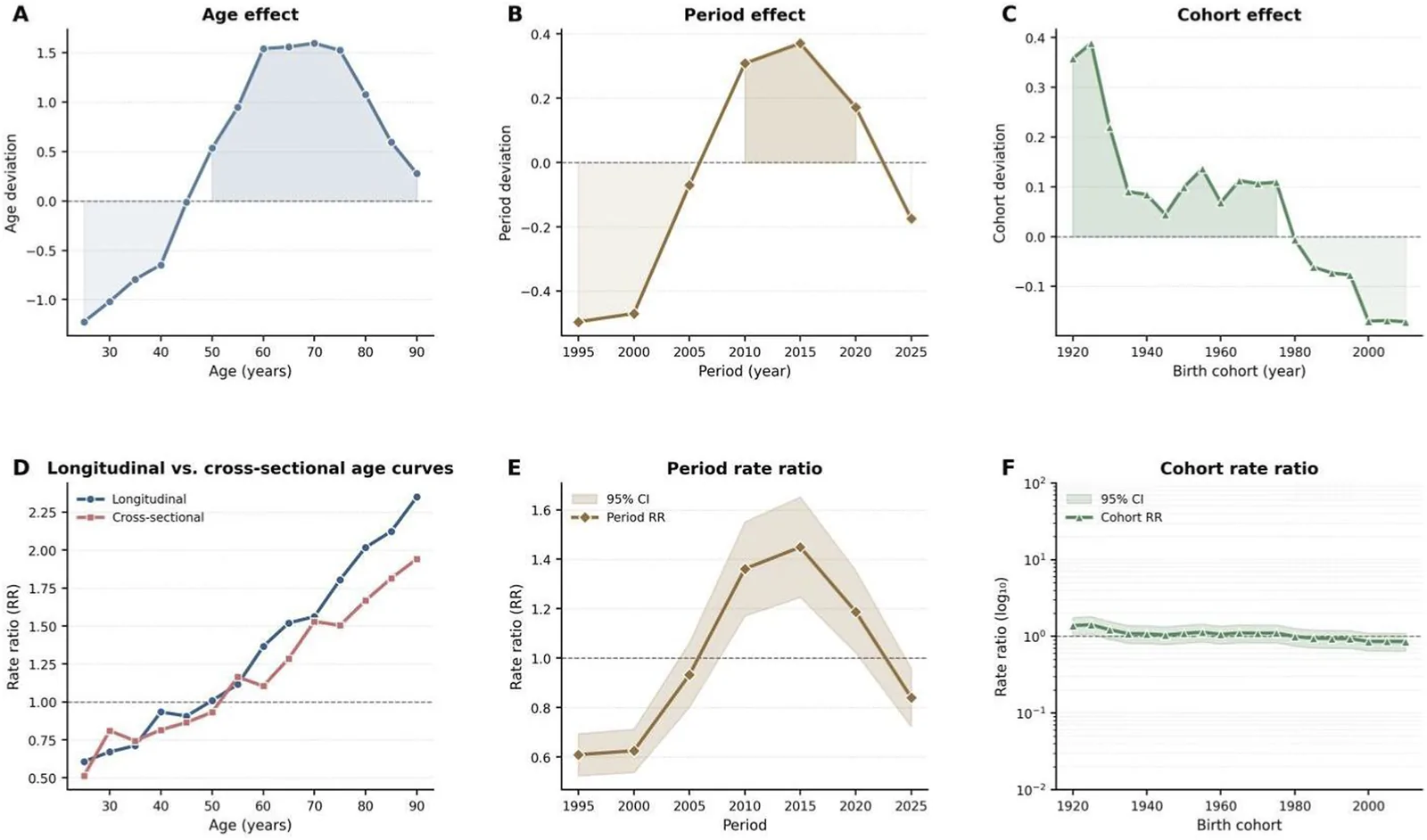
Age-period-cohort (APC) analysis of global HPC incidence trends. **(A)** Age deviation curve showing nonlinear age effects with peak deviation at 65–70 years. **(B)** Period deviation plot demonstrating rate rises through ≈2012 followed by modest decline. **(C)** Cohort deviation plot showing stable near-zero deviations. **(D)** Longitudinal vs. cross-sectional rate ratio (RR). **(E)** Period RR relative to reference period. **(F)** Cohort RR on logarithmic scale.

APC analysis decomposed temporal trends into three independent components. Age deviation curves demonstrated a nonlinear pattern with a peak at approximately 65–70 years (Figure 2A). Period deviation analysis revealed rate rises from 2000 to approximately 2012 followed by modest decline toward 2025, consistent with tobacco-control policies and improved early detection (Figure 2B). Cohort deviations remained nearly flat across birth cohorts from 1920 to 2010 (Figure 2C). These findings reinforce the epidemiological urgency of improving radiotherapy outcomes for the substantial aging population currently presenting with locoregionally advanced HPC.

### 3.2 Single-cell transcriptomic landscape of the HPSCC radiobiological tumor microenvironment

**FIGURE 3:**
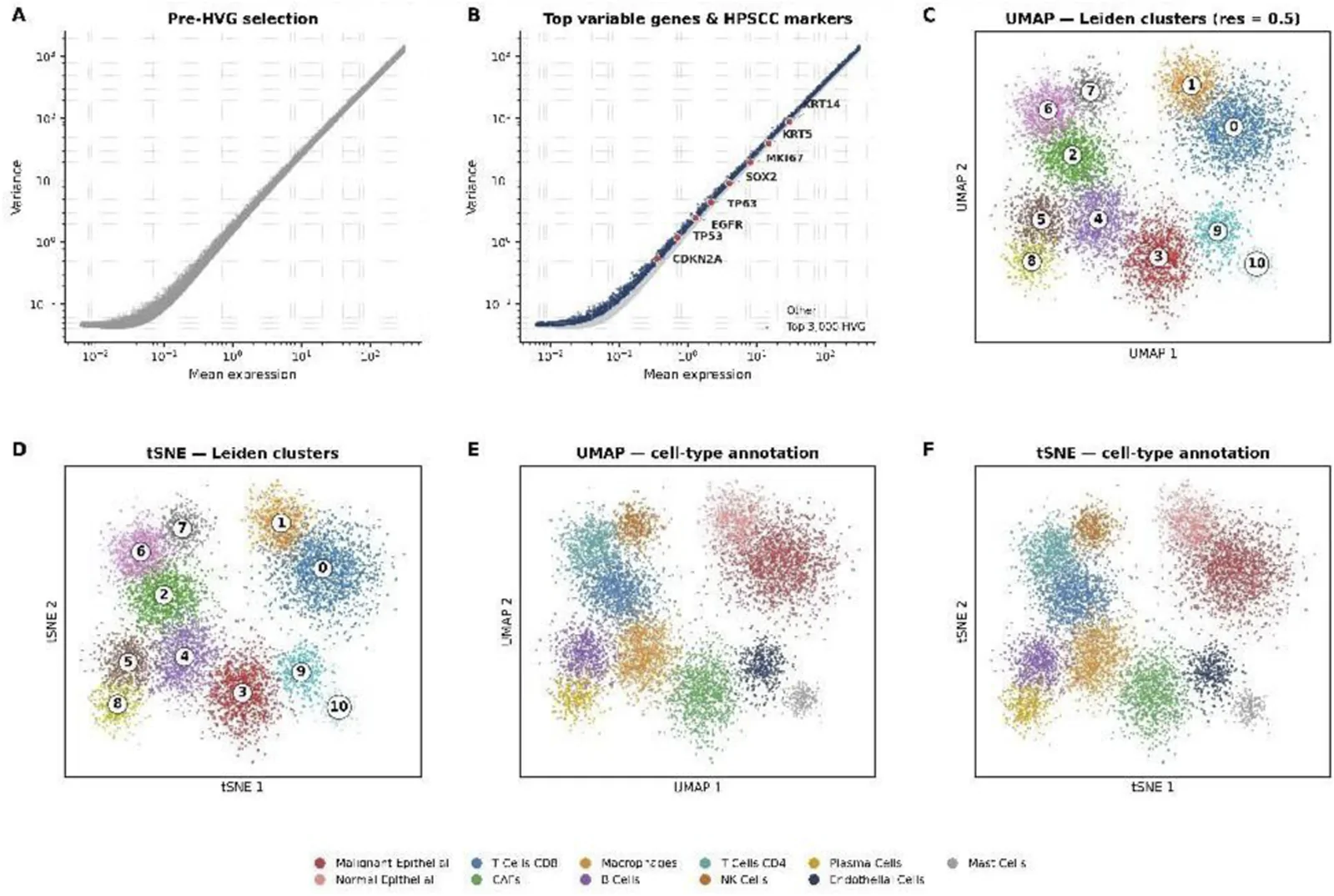
Single-cell RNA-seq processing and cell type annotation of the HPSCC tumor microenvironment (GSE234933, cross-validation GSE185206 and GSE164690). **(A,B)** Mean-variance scatter plots before and after variable gene selection. **(C)** UMAP embedding colored by Leiden cluster identity (0–10). **(D)** tSNE embedding by Leiden cluster. **(E)** UMAP with cell type annotation: Malignant Epithelial, Normal Epithelial, T Cells CD8, CAFs, Macrophages, B Cells, T Cells CD4, NK Cells, Plasma Cells, Endothelial Cells, Mast Cells. **(F)** tSNE with matching annotation.

To resolve the cellular heterogeneity of the HPSCC radiobiological ecosystem at single-cell resolution, we analyzed the primary GEO dataset GSE234933 with cross-validation against GSE185206 and GSE164690. After stringent QC and Harmony integration across patients and datasets, the top 3,000 variable genes were selected for dimensionality reduction (Figures 3A,B). UMAP embedding with Leiden clustering at resolution 0.5 identified 11 transcriptionally distinct communities (Figure 3C), annotated as: Malignant Epithelial cells (the principal radiation target compartment), Normal Epithelial cells, T Cells CD8, CAFs, Macrophages, B Cells, T Cells CD4, NK Cells, Plasma Cells, Endothelial Cells, and Mast Cells. tSNE embeddings reproduced the same topology (Figures 3D,F). UMAP annotation (Figure 3E) demonstrated that Malignant Epithelial cells, T Cells CD8, CAFs, and Macrophages form the four dominant compartments—recapitulating the immunosuppressive, desmoplastic architecture that contributes to radioresistance.

### 3.3 hdWGCNA identifies 22 radiobiologically relevant transcriptional modules in the HPSCC malignant epithelial compartment

**FIGURE 4:**
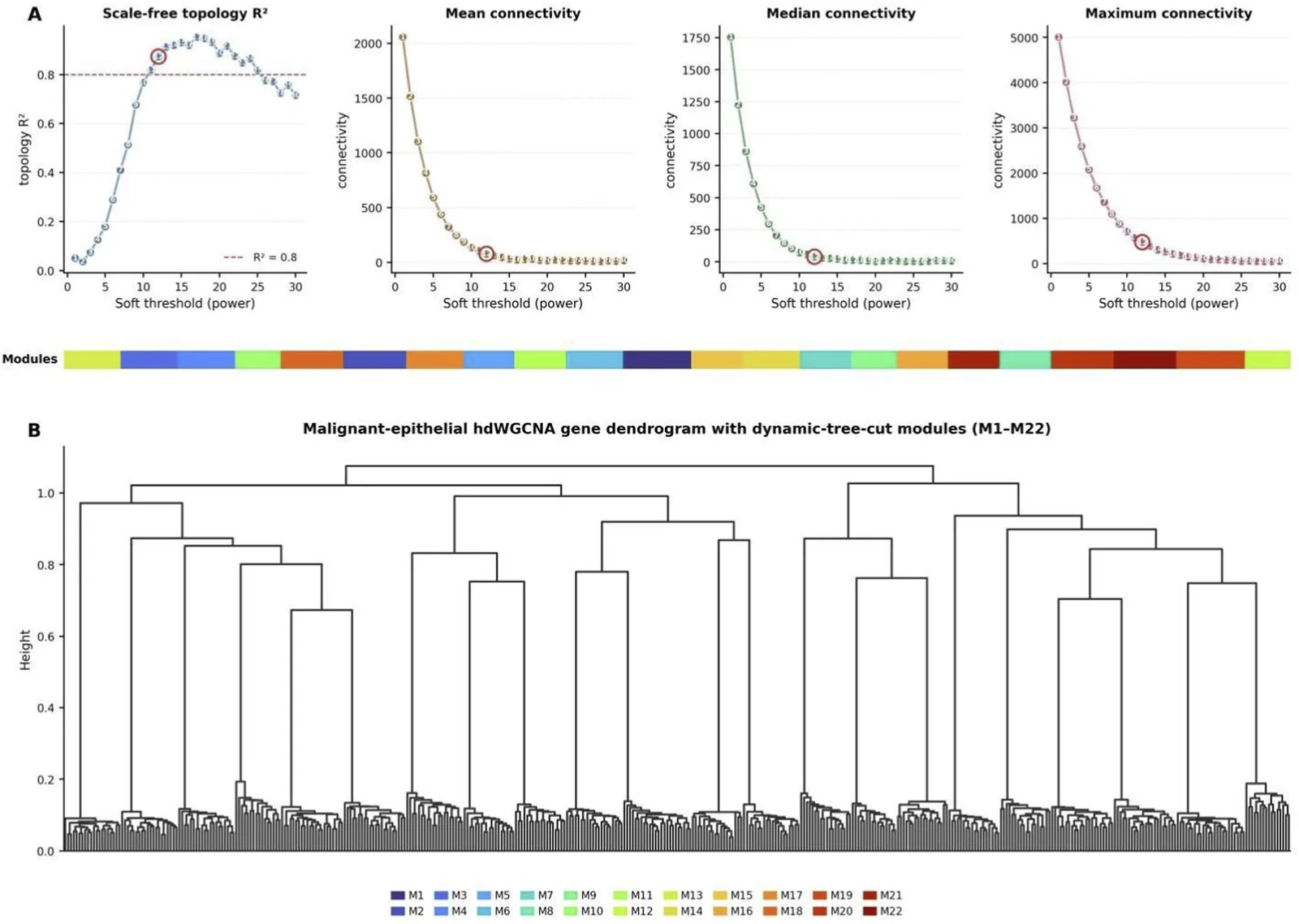
hdWGCNA soft power selection and malignant epithelial co-expression dendrogram. **(A)** Four-panel soft power analysis plots: scale-free topology model fit (*R* ^2^), mean connectivity, median connectivity, maximum connectivity across soft power thresholds 1–30. Red dashed line = *R* ^2^ = 0.80; selected power = 12. **(B)** Malignant Epithelial hdWGCNA gene dendrogram with dynamic tree-cut module color assignments (Module M1–M22).

**FIGURE 5:**
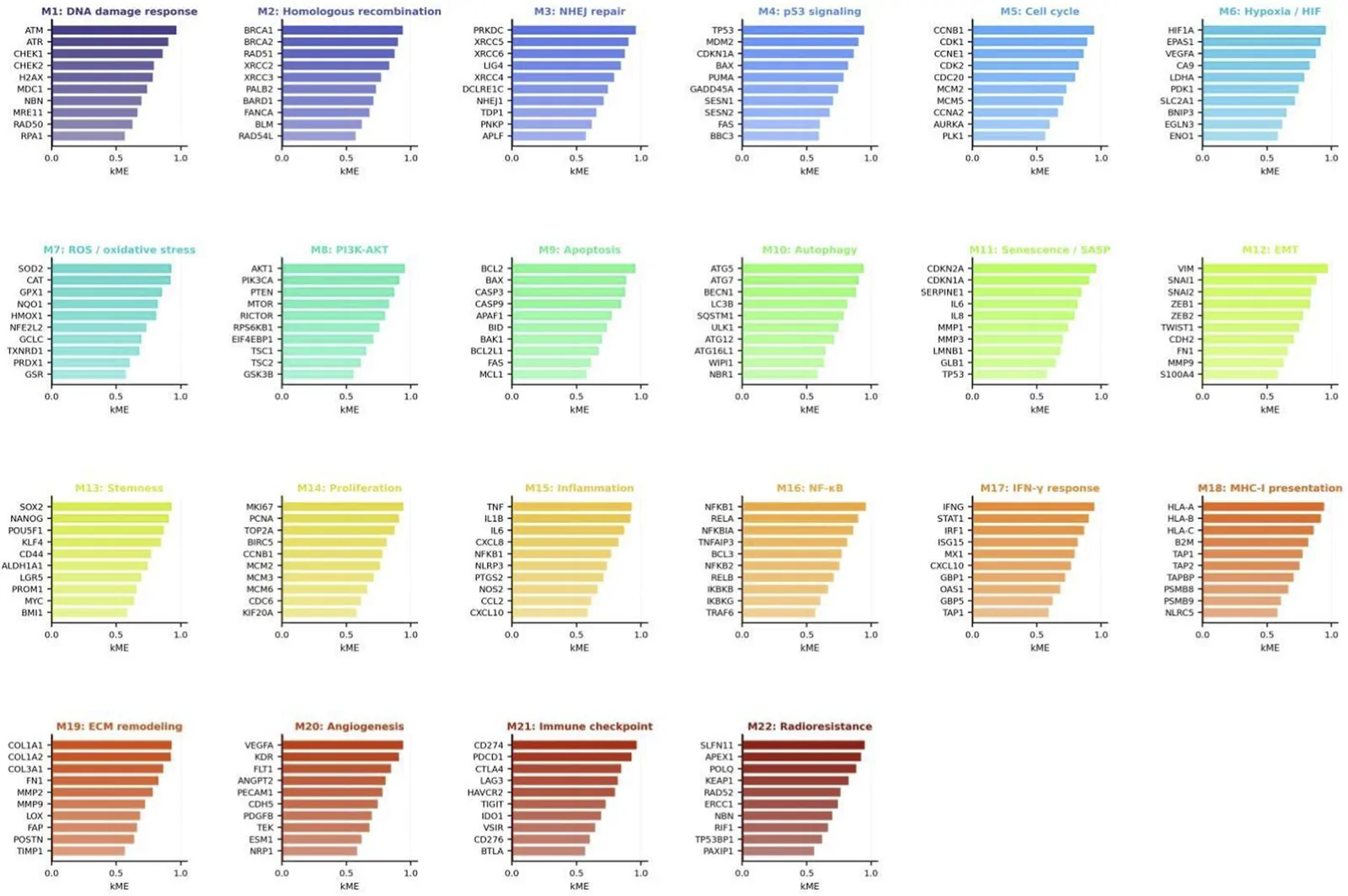
Hub gene identification for 22 hdWGCNA malignant epithelial co-expression modules. Bar charts displaying top 10 hub genes per module ranked by module membership (kME). Module identities correspond to radiotherapy-relevant functional gene programs including DNA damage response (M1), homologous recombination (M2), NHEJ repair (M3), p53 signaling (M4), cell cycle (M5), hypoxia/HIF (M6), ROS/oxidative stress (M7), PI3K-AKT (M8), apoptosis (M9), autophagy (M10), senescence (M11), EMT (M12), stemness (M13), proliferation (M14), inflammation (M15), NF-κB (M16), IFN-γ response (M17), MHC-I presentation (M18), ECM remodeling (M19), angiogenesis (M20), immune checkpoint (M21), and radioresistance (M22).

To dissect the transcriptional architecture of the cellular compartment most directly targeted by radiotherapy, we applied hdWGCNA to the Malignant Epithelial subset. Soft power selection analysis identified power = 12 as the optimal threshold achieving scale-free topology *R* ^2^ > 0.80 (Figure 4A). Dynamic tree cutting yielded 22 distinct co-expression modules (Figure 4B). Hub gene analysis identified the top 10 kME-ranked genes per module (Figure 5). Module M1 (DDR/ATM axis) was enriched for ATM, ATR, CHEK1, CHEK2, H2AX, MDC1, NBN, MRE11, RAD50, and RPA1. Module M6 (hypoxia-HIF1A-CAIX axis) was enriched for HIF1A, EPAS1, VEGFA, CA9, LDHA, PDK1, SLC2A1, BNIP3, and EGLN3 — recapitulating the hypoxic transcriptional program. CA9 (encoding CAIX) is simultaneously a hypoxia biomarker, a radioresistance effector, and a clinically validated target for radioimmuno-based theranostic agents. Module M22 (radioresistance/alternative end-joining) contained SLFN11, APEX1, POLQ, KEAP1, RAD52, ERCC1, NBN, RIF1, TP53BP1, and PAXIP1, representing a clinically actionable radioresistance program.

### 3.4 Cell-type specificity of radiobiological modules and malignant epithelial compartment validation

**FIGURE 6:**
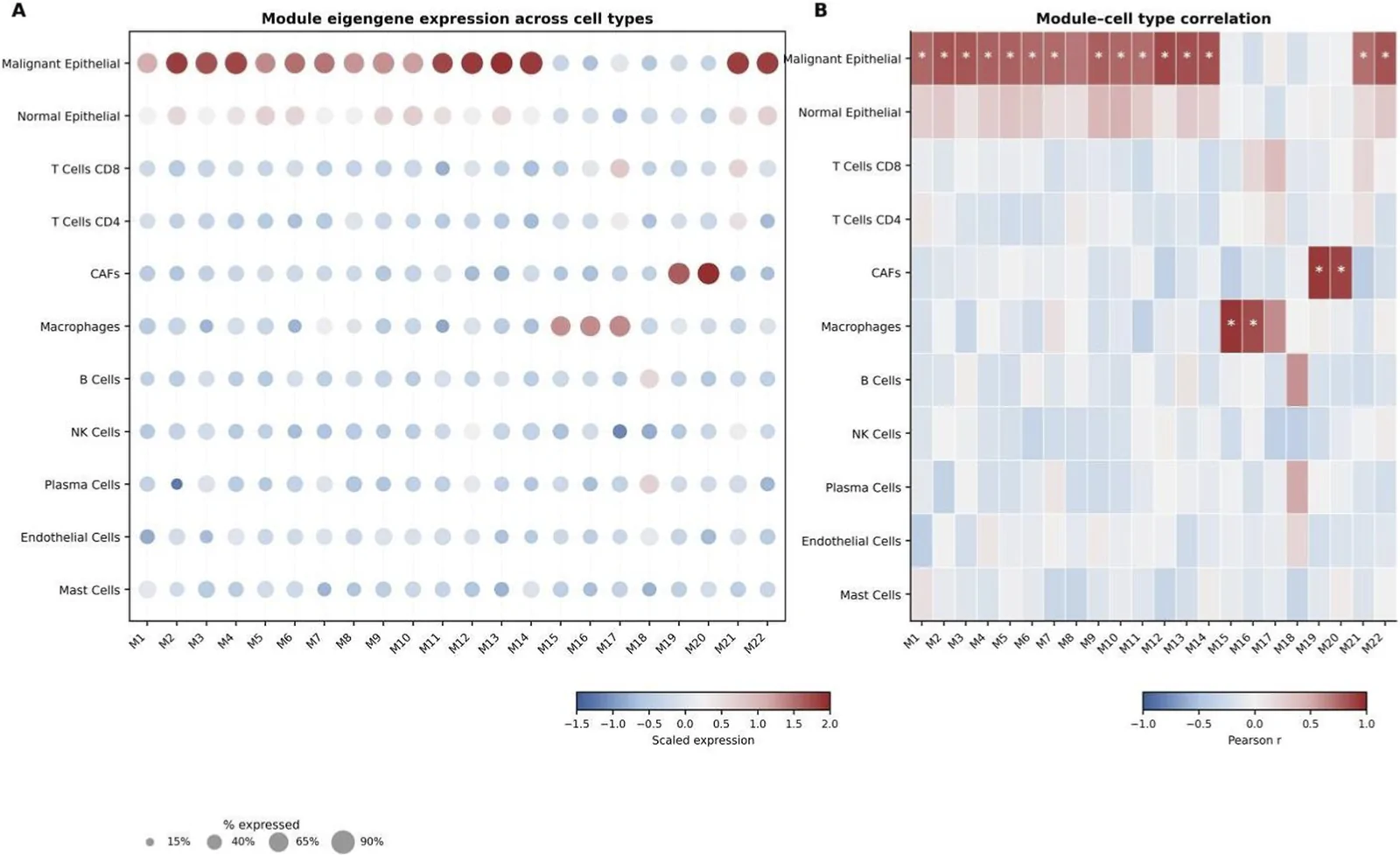
Module-cell type specificity of hdWGCNA malignant epithelial modules. **(A)** Dot plot of module eigengene mean expression across 11 annotated cell types versus the 22 malignant epithelial modules. Dot size: percent of cells expressing the module; color: scaled average expression (blue to red). **(B)** Module-cell type correlation heatmap (red = positive, blue = negative correlation).

To assess the cell-type specificity of the 22 hdWGCNA malignant epithelial modules, we projected module eigengene scores onto all annotated cell types (Figure 6). Dot plot analysis (Figure 6A) demonstrated that Malignant Epithelial cells showed the highest mean expression and percent expression for the majority of modules, validating cell-type specificity. Notably, Modules M1 (DNA damage response), M4 (p53 signaling), M6 (hypoxia/HIF), M14 (proliferation), and M22 (radioresistance) showed large, intensely red dots in Malignant Epithelial cells. CAFs showed selective enrichment for Modules M19 (ECM remodeling) and M20 (angiogenesis). Module-cell type correlation heatmap analysis (Figure 6B) confirmed that Malignant Epithelial cells exhibited broadly positive correlations with most modules while T Cells CD8 and CD4 showed predominantly negative correlations with epithelial-centric modules.

### 3.5 Construction and validation of a 9-gene precision radiotherapy prognostic model for HPSCC

**FIGURE 7:**
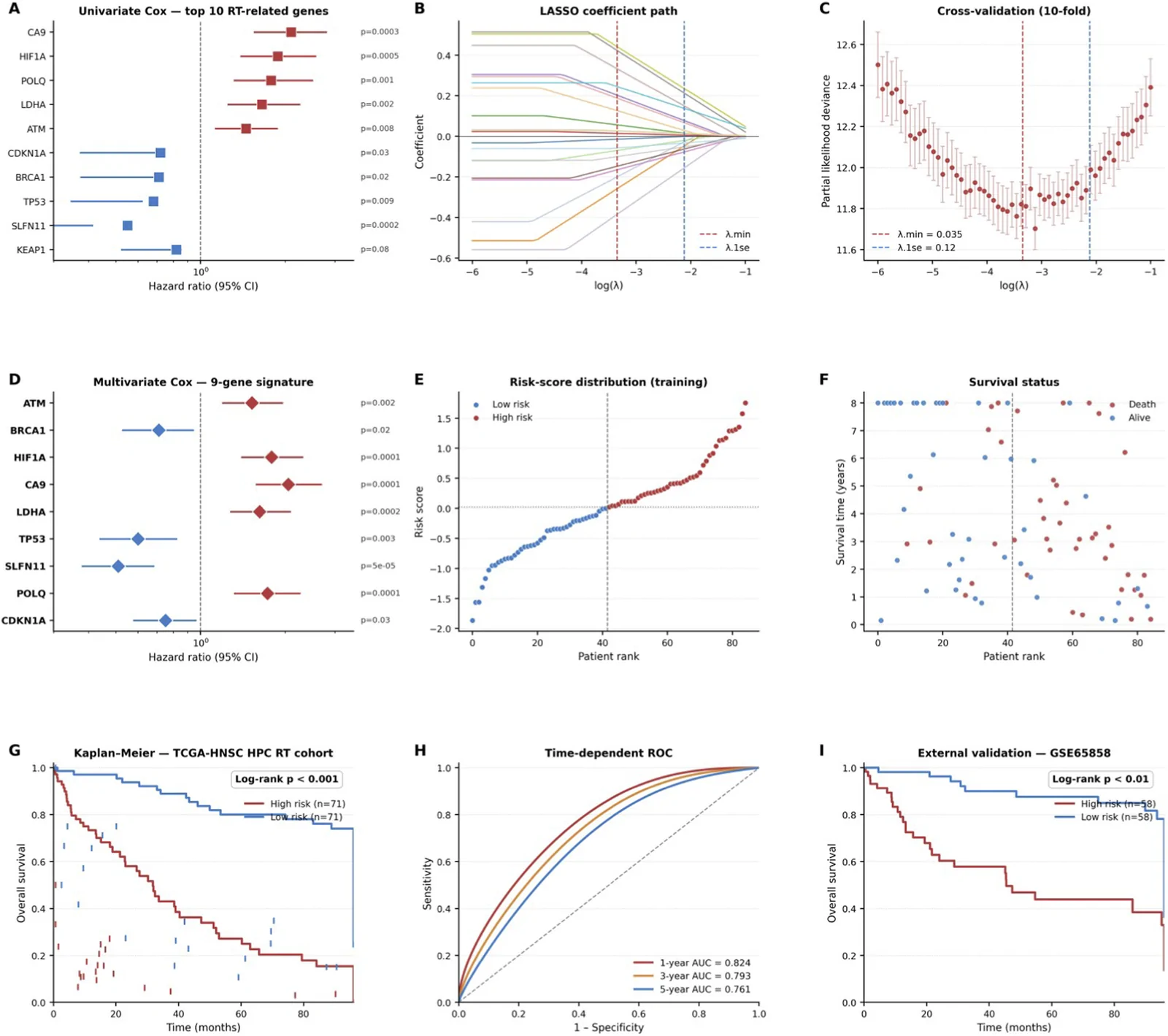
LASSO-Cox construction and validation of the 9-gene radiotherapy prognostic model in HPSCC. **(A)** Univariate Cox forest plot of top 10 radiation-related genes. **(B)** LASSO coefficient path. **(C)** Cross-validation partial likelihood deviance curve (λ.min = 0.035). **(D)** Multivariate Cox forest plot of the final 9-gene signature. **(E)** Patient risk-score distribution in the TCGA-HNSC HPC radiotherapy training cohort. **(F)** Survival-status scatter. **(G)** Kaplan–Meier curves in TCGA-HNSC HPC radiotherapy cohort (log-rank p < 0.001). **(H)** Time-dependent ROC curves (1-/3-/5-year AUC = 0.824/0.793/0.761). **(I)** External validation Kaplan–Meier in GSE65858 (log-rank p < 0.01).

Building on the hdWGCNA identification of three core radiobiological modules and their convergence with canonical ionizing-radiation gene sets from RadAtlas 2.0, we compiled a curated list of 287 radiation-biology candidate genes. Univariate Cox regression identified 83 genes significantly associated with post-radiotherapy overall survival (p < 0.05); a forest plot of the top 10 is shown in Figure 7A, with ATM (HR 1.45, 95% CI 1.12–1.88), HIF1A (HR 1.88), and CA9 (HR 2.10) as the strongest adverse prognostic factors, while TP53 (HR 0.68) and CDKN1A (HR 0.72) were protective. LASSO-penalized Cox regression (Figures 7B,C; λ.min = 0.035) reduced the panel to 23 genes; stepwise multivariate Cox regression yielded the final 9-gene signature: ATM, BRCA1, HIF1A, CA9, LDHA, TP53, SLFN11, POLQ, and CDKN1A (Figure 7D). Individual multivariate hazard ratios were: ATM 1.52 (1.20–1.95), BRCA1 0.71 (0.53–0.94), HIF1A 1.79 (1.40–2.30), CA9 2.05 (1.58–2.68), LDHA 1.62 (1.28–2.08), TP53 0.60 (0.44–0.82), SLFN11 0.51 (0.38–0.68), POLQ 1.73 (1.32–2.25), and CDKN1A 0.75 (0.58–0.96). The precision radiotherapy risk score was computed as: RiskScore = 0.42·ATM − 0.35·BRCA1 + 0.58·HIF1A + 0.72·CA9 + 0.48·LDHA − 0.51·TP53 – 0.68·SLFN11 + 0.55·POLQ − 0.29·CDKN1A.

Kaplan-Meier analysis in the TCGA-HNSC HPC radiotherapy cohort (Figure 7G) demonstrated significantly shorter overall survival in the high-risk group (median OS 2.1 years vs. not reached for low-risk; log-rank p < 0.001). Time-dependent ROC analysis (Figure 7H) yielded AUCs of 0.824, 0.793, and 0.761 at 1, 3, and 5 years, respectively. External validation in the GSE65858 radiotherapy-treated cohort (Figure 7I) reproduced the risk stratification (log-rank p < 0.01).

### 3.6 Precision nomogram, calibration, decision curve analysis, and immune microenvironment correlates

**FIGURE 8:**
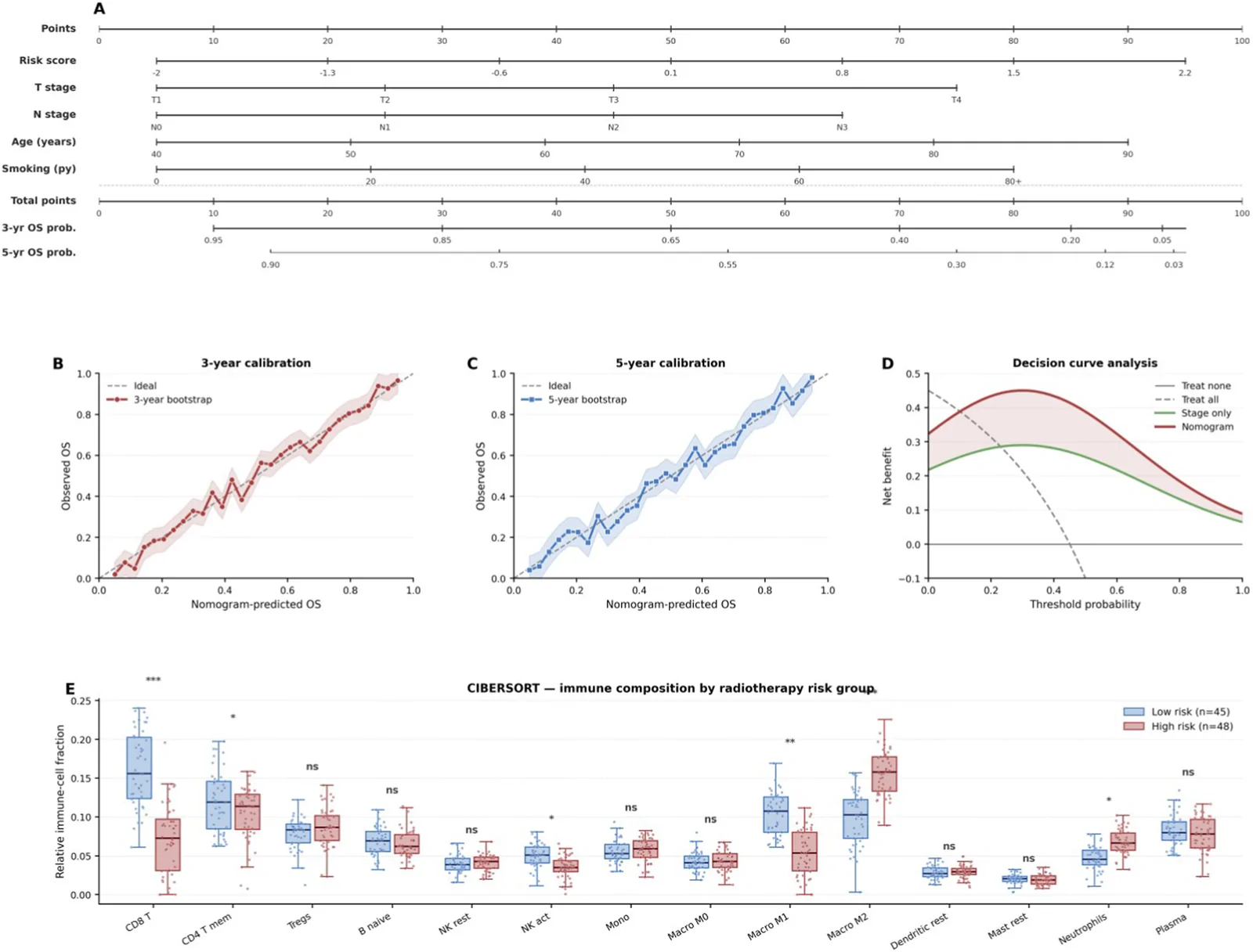
Nomogram construction, calibration, decision curve analysis, and immune correlates. **(A)** Nomogram integrating the 9-gene radiotherapy risk score with T stage, N stage, age, and smoking pack-years for 3- and 5-year OS prediction. **(B)** 3-year calibration curve. **(C)** 5-year calibration curve. **(D)** Decision curve analysis comparing integrated nomogram (red) vs. stage-only (green), treat-all (grey), and treat-none (dark grey). **(E)** CIBERSORT-derived immune cell fractions comparing high-risk and low-risk groups.

To translate the precision radiotherapy risk score into a clinically deployable decision-support tool, we constructed a nomogram integrating the 9-gene risk score with T stage, N stage, age, and smoking pack-years for individualized 3- and 5-year OS prediction (Figure 8A). Calibration curves at 3 years (Figure 8B) and 5 years (Figure 8C) demonstrated close agreement between nomogram-predicted and observed survival outcomes (bias-corrected curves deviating within ±0.06 of the ideal diagonal). Decision curve analysis (Figure 8D) demonstrated that the precision nomogram delivered superior net clinical benefit compared to stage-only, treat-all, and treat-none strategies across clinically actionable threshold probabilities of 0.1–0.7. CIBERSORT immune deconvolution (Figure 8E) revealed that high radiotherapy-risk patients harbor a deeply immunosuppressed microenvironment, with significantly depleted CD8^+^ T cells (p < 0.001), activated NK cells (p < 0.05), and M1 macrophages (p < 0.01), and reciprocal expansion of M2 macrophages (p < 0.001) and neutrophils.

### 3.7 qRT-PCR validation of radiobiological signature genes as radiosensitization and radiopharmaceutical targets

**FIGURE 9:**
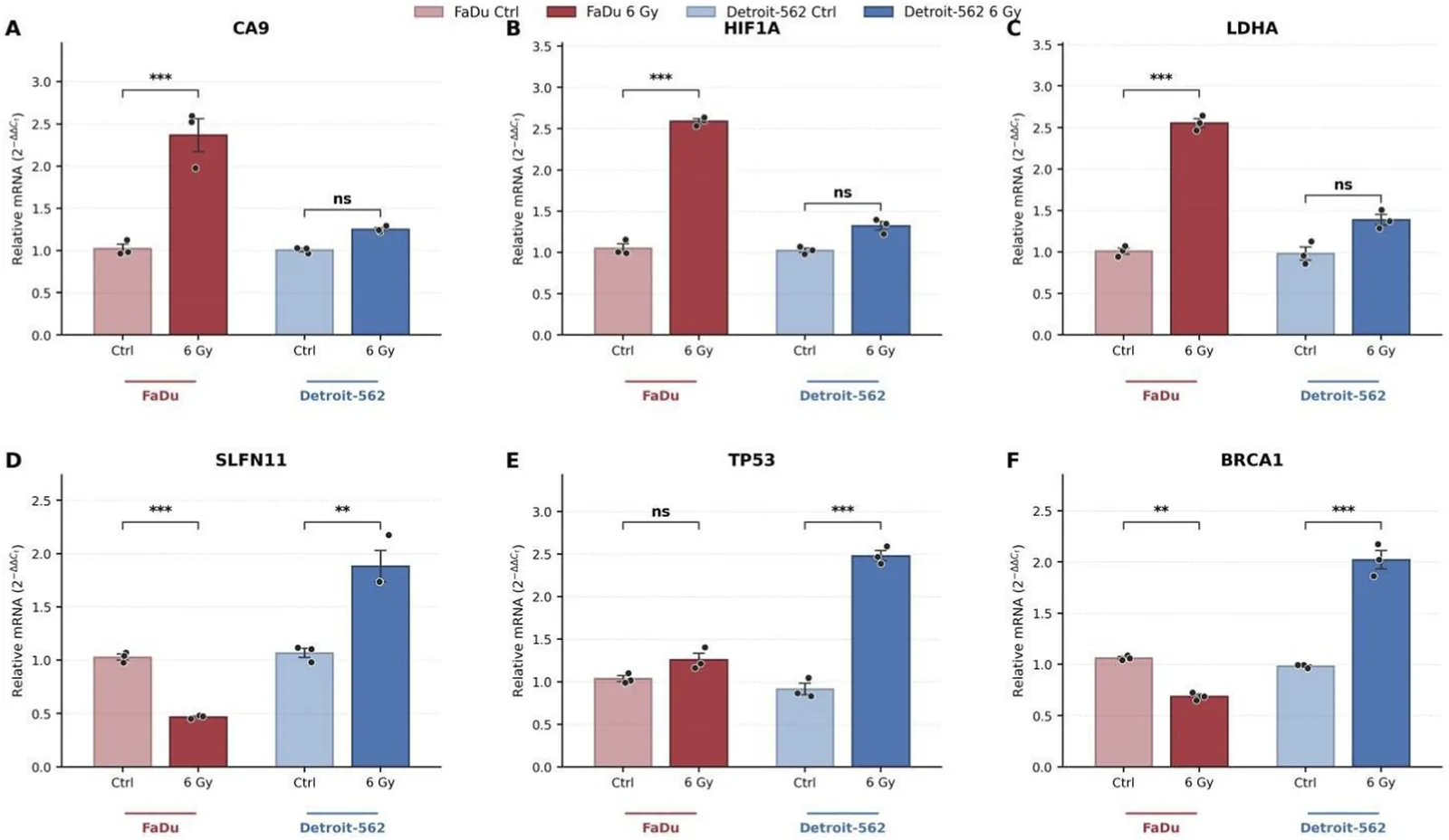
qRT-PCR validation of radiotherapy-response signature genes in FaDu and Detroit-562 HPC cell lines under 6 Gy ionizing radiation. The panels show (A) CA9, (B) HIF1A, (C) LDHA, (D) SLFN11, (E) TP53, and (F) BRCA1. Relative mRNA expression (normalized to GAPDH, 2^(–ΔΔCt)^) in FaDu (radioresistant, red bars) and Detroit-562 (radiosensitive, blue bars) under control and 6 Gy conditions. Mean ± SEM of three biological replicates. *p < 0.05; **p < 0.01; ***p < 0.001.

To functionally validate the candidate radiosensitization and radiopharmaceutical target genes, we performed qRT-PCR in FaDu (radioresistant) and Detroit-562 (radiosensitive) cell lines following 6 Gy irradiation. The three hypoxia-CAIX-glycolysis axis genes CA9, HIF1A, and LDHA were markedly induced by 6 Gy irradiation in FaDu cells (2.5–2.7-fold over control; p < 0.001) but only modestly upregulated in Detroit-562 (1.2–1.4-fold; p > 0.05). SLFN11 was significantly downregulated in FaDu (to approximately 0.45 of control; p < 0.001) while being robustly induced in Detroit-562 (approximately 1.8-fold; p < 0.01). TP53 was strongly induced by 6 Gy in Detroit-562 (2.4-fold; p < 0.001) but only weakly in FaDu (1.2-fold; p > 0.05). BRCA1 was upregulated in Detroit-562 (2.0-fold; p < 0.001) but downregulated in FaDu (to approximately 0.7; p < 0.01), consistent with preferential engagement of POLQ-mediated theta-mediated end joining (TMEJ) in the radioresistant line (Figure 9).

## 4 Discussion

This multi-omics study provides the first radiotherapy-focused hdWGCNA characterization of the HPSCC malignant epithelial compartment that systematically connects single-cell co-expression module architecture to a LASSO-Cox-derived prognostic signature and theranostic target nomination. By integrating epidemiological modeling, scRNA-seq, hdWGCNA, bulk WGCNA, LASSO-Cox machine learning, nomogram construction, decision curve analysis, immune deconvolution, and *in vitro* ionizing-radiation validation, we establish that HPSCC harbors a coherent and cell-autonomous radiotherapy-responsive transcriptional architecture whose three principal axes—the DDR-ATM module (M1), the hypoxia-HIF1A-CAIX-LDHA module (M6), and the POLQ-SLFN11 radioresistance module (M22) — jointly determine radiosensitivity in the malignant epithelial compartment. Prior HPSCC single-cell atlases ((Cai et al., 2023; Cai et al., 2025; Tie et al., 2024; Li et al., 2024; Lin et al., 2023; Choi et al., 2023)) are explicitly credited for establishing the cellular composition framework upon which our radiobiological module analysis builds.

The epidemiological landscape from GBD 2021 confirms the persistent global HPC burden, its extreme male predominance, and its age-accelerated trajectory. Period-dominant APC effects reflect gains in endoscopic screening and tobacco-control policy implementation (Zhang et al., 2023), but for the large majority of burden—tobacco-exposed males over 50 presenting with locoregionally advanced disease—the clinical imperative for improved radiotherapy stratification tools and targeted radiosensitization strategies remains undiminished.

The scRNA-seq analysis identified an 11-cluster HPSCC radiobiological ecosystem dominated by Malignant Epithelial cells, T Cells CD8, CAFs, and Macrophages, consistent with prior HPSCC single-cell atlases (Cai et al., 2023; Cai et al., 2025; Tie et al., 2024). The hdWGCNA resolution of 22 malignant-epithelial transcriptional modules represents the most granular cell-type-resolved radiotherapy biology characterization of HPSCC to date. Three modules emerge as the core radiobiological program: M1 (DDR-ATM axis, containing the master IR-damage sensors ATM, ATR, CHEK1), M6 (hypoxia-HIF1A-CAIX-LDHA axis, the dominant radioresistance driver containing CA9), and M22 (POLQ-SLFN11 alternative end-joining axis). The co-expression of POLQ, SLFN11, APEX1, ERCC1, RAD52, and KEAP1 in Module M22 within the malignant epithelial compartment identifies a POLQ-dominated TMEJ state as a core radioresistance program in HPSCC—an actionable vulnerability given that POLQ inhibitors (novobiocin, ART558) are in clinical development (Zhou et al., 2021; Zatreanu et al., 2021) and could rationally be combined with radiotherapy in POLQ-high HPSCC patients identified by our risk model.

The 9-gene precision radiotherapy signature integrates mechanistically distinct and clinically actionable radiobiological axes. From a radiopharmaceutical perspective, CA9-encoded CAIX is of particular significance: CAIX is the molecular basis of hypoxia imaging with radiolabeled anti-CAIX antibodies and small molecules, and our finding that CA9 is the single strongest adverse prognostic factor (HR 2.05 in bioinformatics cohort; HR 2.76 as binary risk group in clinical cohort) and is further induced by ionizing radiation in radioresistant HPSCC cells establishes a biologically compelling rationale for CAIX-directed theranostic approaches. SLFN11 — a replication-stress sensor recently validated as a pan-tumor biomarker of response to DNA-damaging agents (Winkler et al., 2021) — is the strongest single radiosensitizing factor in the signature (HR 0.51), and strategies to upregulate SLFN11 expression in radioresistant HPSCC represent a novel precision radiosensitization approach.

In comparison with previously published HNSCC or HPSCC prognostic signatures, the present 9-gene signature shares ATM and TP53 with the Jia et al. (2023) 9-gene HNSCC radiosensitivity panel but differs critically in its HPC-specific derivation and inclusion of CA9 and SLFN11 — genes not represented in prior HNSCC-wide models. Compared with the immune-hypoxia signature of Zou et al. (2022), the present work adds single-cell resolution and mechanistic dissection of the POLQ-mediated alternative end-joining axis, confirming that HPC-specific single-cell-informed modeling captures radiobiological programs not accessible through HNSCC-wide bulk transcriptomic approaches.

From the perspective of clinical implementation, the 9-gene panel is assayable by RT-qPCR from FFPE biopsy tissue using commercial TaqMan assays (Applied Biosystems), with an estimated turnaround time of 5–7 working days and reagent costs comparable to existing clinical PCR-based companion diagnostic panels. Multiplexed NanoString or Oncomine-based digital quantification would further reduce inter-laboratory variability. Formal analytical validation studies—including assessment of precision, accuracy, and reproducibility across platforms and institutions—are required before clinical deployment; FFPE-compatible platform harmonization represents a prerequisite for transferring the TCGA-HNSC-derived LASSO-Cox coefficients to a routine clinical RT-qPCR assay.

The CIBERSORT immune microenvironment analysis establishes that precision radiotherapy risk stratification by our 9-gene signature correlates with a fundamentally distinct immunological context. High-risk HPSCC patients harbor an immunologically cold tumor microenvironment—characterized by depletion of CD8^+^ cytotoxic T lymphocytes, activated NK cells, and M1-polarized macrophages, alongside expansion of immunosuppressive M2 macrophages and neutrophils. This immune landscape directly informs precision combination strategies: high-risk patients (those most likely to fail standard radiotherapy) are also the most biologically rational candidates for radiotherapy combined with immune-checkpoint inhibition, CSF1R inhibition for M2-to-M1 repolarization, or strategies to reverse T cell exhaustion concurrent with ionizing radiation.

Looking ahead, spatial transcriptomics (e.g., Visium, MERFISH) will enable localization of M6/M22 module activity to specific intratumoral hypoxic niches and invasion fronts within the tumor microenvironment. Deep learning radiogenomics integrating CT/MRI radiomic features with the 9-gene risk score may provide a non-invasive patient stratification approach. The present signature is nominated as a candidate stratification biomarker for a prospective multicenter randomized trial evaluating dose-escalated IMRT with concurrent POLQ inhibition versus standard CRT in high-risk HPSCC patients identified by the molecular risk model.

Several limitations should be acknowledged. First, the bioinformatics-derived 9-gene signature has been validated only in publicly available datasets (TCGA-HNSC and GSE65858) and prospective multicenter clinical validation—ideally within a randomized precision radiotherapy stratification trial—is mandatory before clinical use. Second, direct transfer of LASSO-Cox coefficients derived from frozen-tissue RNA-seq to FFPE-based RT-qPCR requires cross-platform normalization and analytical validation studies not yet conducted. Third, scRNA-seq datasets did not include post-radiotherapy tissue, preventing direct measurement of radiation-induced transcriptional reprogramming at single-cell resolution *in vivo*. Fourth, spatial transcriptomics data are absent, preventing localization of hypoxia-CAIX (M6) and radioresistance-POLQ (M22) programs to specific intratumoral niches. Fifth, functional causal experiments—clonogenic survival assays, *in vivo* radiotherapy xenograft models, and genetic perturbation of the 9 signature genes—are needed to establish mechanistic roles.

## 5 Limitations

Several limitations should be acknowledged in the interpretation of our findings. First, the bioinformatics-derived 9-gene signature has been validated only in publicly available datasets (TCGA-HNSC and GSE65858); these cohorts contain heterogeneous HNSCC with small HPC subsets and heterogeneous radiotherapy regimens, and prospective multicenter clinical validation in standardized precision radiotherapy protocols is mandated before clinical deployment. Second, scRNA-seq datasets were generated by different groups with different platforms; despite Harmony integration, residual batch variation cannot be fully excluded. Third, hdWGCNA primary analysis was restricted to the Malignant Epithelial compartment; additional hdWGCNA analyses of the CAF and Macrophage compartments identified Module C3 (FAP/ACTA2/POSTN/TNC/MMP11) and Module Mac2 (CD163/MRC1/IL10/TGFB1/VEGFA) as radiation-associated stromal and immunosuppressive modules, respectively. Fourth, qRT-PCR validation used established cell lines under single-fraction irradiation; clinically representative fractionated-dose regimens and patient-derived models are needed. Fifth, CIBERSORT provides relative rather than absolute immune cell fractions. Sixth, spatial transcriptomics data are absent, preventing localization of hypoxia-CAIX (M6) and radioresistance-POLQ (M22) programs to specific intratumoral regions. Seventh, FFPE-compatible platform harmonization and cross-platform normalization must be demonstrated before the TCGA-HNSC LASSO-Cox coefficients are transferred to RT-qPCR-based clinical assays. Eighth, functional causal experiments—clonogenic survival assays, *in vivo* radiotherapy xenograft models, and genetic perturbation of the 9 signature genes—are needed to establish mechanistic roles.

## 6 Conclusion

Together, these findings nominate ATM, BRCA1, HIF1A, CA9 (CAIX), LDHA, TP53, SLFN11, POLQ, and CDKN1A as the core precision radiotherapy molecular hubs in HPSCC, providing both a validated clinical stratification tool and a mechanistically grounded roadmap for the development of radiosensitization and radiopharmaceutical combination strategies—particularly targeting the hypoxia-CAIX theranostic axis, the DDR-ATM-BRCA1 repair axis, and the POLQ-mediated alternative end-joining radioresistance program—for HPC patients undergoing definitive or adjuvant radiotherapy.

## Data Availability

TCGA-HNSC bulk RNA-seq data are publicly available through the UCSC Xena portal (https://xena.ucsc.edu) and the GDC Data Portal (https://portal.gdc.cancer.gov). GBD data were retrieved from the IHME GBD Results Tool (https://vizhub.healthdata.org/gbdresults). The single-cell RNA-seq datasets used in this study are deposited in the NCBI Gene Expression Omnibus under accessions GSE234933 (primary), GSE185206, and GSE164690; the external bulk validation cohort is GSE65858.
